# Microbiome Dynamics in a Shrimp Grow-out Pond with Possible Outbreak of Acute Hepatopancreatic Necrosis Disease

**DOI:** 10.1038/s41598-017-09923-6

**Published:** 2017-08-24

**Authors:** Wei-Yu Chen, Tze Hann Ng, Jer-Horng Wu, Jiung-Wen Chen, Han-Ching Wang

**Affiliations:** 1 Department of Environmental Engineering, National Cheng Kung University, Tainan, 70101 Taiwan (ROC); 2 Department of Biotechnology and Bioindustry Sciences, National Cheng Kung University, Tainan, 70101 Taiwan (ROC); 3 Center for Shrimp Disease Control and Genetic Improvement, National Cheng Kung University, Tainan, 70101 Taiwan (ROC)

## Abstract

Acute hepatopancreatic necrosis disease (AHPND) (formerly, early mortality syndrome) is a high-mortality-rate shrimp disease prevalent in shrimp farming areas. Although AHPND is known to be caused by pathogenic *Vibrio parahaemolyticus* hosting the plasmid-related PirAB^vp^ toxin gene, the effects of disturbances in microbiome have not yet been studied. We took 62 samples from a grow-out pond during an AHPND developing period from Days 23 to 37 after stocking white postlarvae shrimp and sequenced the 16S rRNA genes with Illumina sequencing technology. The microbiomes of pond seawater and shrimp stomachs underwent varied dynamic succession during the period. Despite copies of PirAB^vp^, principal co-ordinates analysis revealed two distinctive stages of change in stomach microbiomes associated with AHPND. AHPND markedly changed the bacterial diversity in the stomachs; it decreased the Shannon index by 53.6% within approximately 7 days, shifted the microbiome with *Vibrio* and *Candidatus* Bacilloplasma as predominant populations, and altered the species-to-species connectivity and complexity of the interaction network. The AHPND-causing *Vibrio* species were predicted to develop a co-occurrence pattern with several resident and transit members within *Candidatus* Bacilloplasma and *Cyanobacteria*. This study’s insights into microbiome dynamics during AHPND infection can be valuable for minimising this disease in shrimp farming ponds.

## Introduction

Acute hepatopancreatic necrosis disease/syndrome (AHPND/S) [formerly called early mortality syndrome (EMS)] is an emerging shrimp disease. Since the first case reported in southern China in 2009, AHPND has also been observed in shrimp farms in other areas of Asia and America, and the outbreak has inflicted serious damage on the global shrimp farming industry with annual losses of more than US$1 billion^1, 2^. The disease typically affects postlarvae/juvenile shrimp within 20–35 days of stocking a fresh rearing pond and causes progressive degeneration of the hepatopancreas, including sloughing of the hepatopancreas tubule epithelial cells in the early stage and necrosis and massive hemocytic infiltration in the terminal stage^3, 4^. A high mortality rate (40–100%) occurs in a shrimp cultivation pond upon AHPND infection^5^. AHPND is caused by insecticidal *Photorhabdus* insect-related (Pir) binary toxins produced by the PirAB^vp^ genes in the pVA1 plasmid^6^, which is specifically hosted by several strains of *Vibrio parahaemolyticus* in the *Vibrio harveyi* clade^4^. Although several efforts have been made to reduce the number of pathogenic Vibrio bacteria in shrimp farms to minimize AHPND outbreaks, AHPND is still not under control in many areas^7^.

The health of an animal is highly associated with the composition and function of a microbial community (referred to as microbiome) inside that animal’s intestinal system. Microbiome studies of terrestrial animals have revealed cause-and-effect relationships and have suggested that the ‘health’ of animal microbiome influences the colonization, growth, and virulence of invading pathogens^8, 9^. However, very few studies have investigated aquatic animals, and most of these have focused on fish species rather than crustaceans such as shrimp. Actually, a shrimp farming pond is an aquatic ecosystem where planktonic microbes proliferate with shrimp larvae. In a pond with daily receipt of intensive nutrients from feeding fodder and faecal matter produced by shrimp, the water microbiome usually evolves with the water quality over time^10^. Thus, the emergence of disease in pond water is believed to result from complex interactions between the host, water quality parameters, and the activities of the corresponding microbial populations^11^.

Vibrio bacteria comprising many opportunistic pathogen species are characterized as fast-growing aquatic microorganisms (*r* strategists)^12^. Their growth usually benefits from the eutrophication of pond water and may lead to colonization of the digestive system, causing gastrointestinal diseases in sea animals^13^. Our previous study demonstrated that an AHPND-causing *V*. *parahaemolyticus* strain initially colonized and replicated in the stomach and then appeared in the hepatopancreas^14^. This raises several questions. What are the healthy and diseased stomach microbiomes in penaeid shrimp? How do stomach microbiomes change from healthy to diseased states? To what extent do water and stomach microbiomes interact with each other? What is the abundance and dynamic of pathogenic *V*. *parahaemolyticus* and what are its interactions with other bacterial populations in the stomachs of animals with AHPND? Because development and maintenance of healthy intestinal microbiomes are critical in resisting pathogenic infections, the underlying microbial ecology of the farming pond must be investigated for developing strategies and products to control this disease.

Cost-effective and powerful high-throughput sequencing techniques have been developed to identify large-scale microbial phylotypes and to detect rare taxa in samples. These techniques have been applied to study bacterioplankton during shrimp cultivation^15^, bacterial community structures in the intestines of shrimp at different growth stages^16^, effects of environmental factors on the microbial community in culturing tanks^17^, effects of lipid sources on intestinal microbiota^18^, the bacterial community associated with the intestinal tract of shrimp^19, 20^, and effects of host phylogeny and habitats on the gut microbiome of shrimp^21^. Previous studies have investigated the microbial communities of healthy shrimp sampled from a single medium (pond water or shrimp intestine) at a given point in time. However, most studies analysed samples from the shrimp midgut and hindgut (i.e., intestine) rather than from the foregut (i.e., stomach). A study of the microbiome in the stomach can aid in understanding the ecological mechanism associated with AHPND because of the close proximity of the stomach and hepatopancreas. So far, time-series bacterial data have not been available, especially for both shrimp and pond water sampled in a shrimp farming pond during an outbreak of AHPND.

In this study, over several days, bacterial samples of pond seawater and the stomachs of shrimp with or without AHPND were obtained from a commercial shrimp farming pond, which ultimately succumbed to an outbreak of AHPND. The samples were studied using the high-throughput 16S rRNA sequencing approach. The present study elucidates the dynamics and interaction between microbiomes of pond seawater and shrimp stomachs in association with AHPND.

## Results

### Monitoring of water quality, 16S rRNA gene, and toxin gene

**Figure 1:**
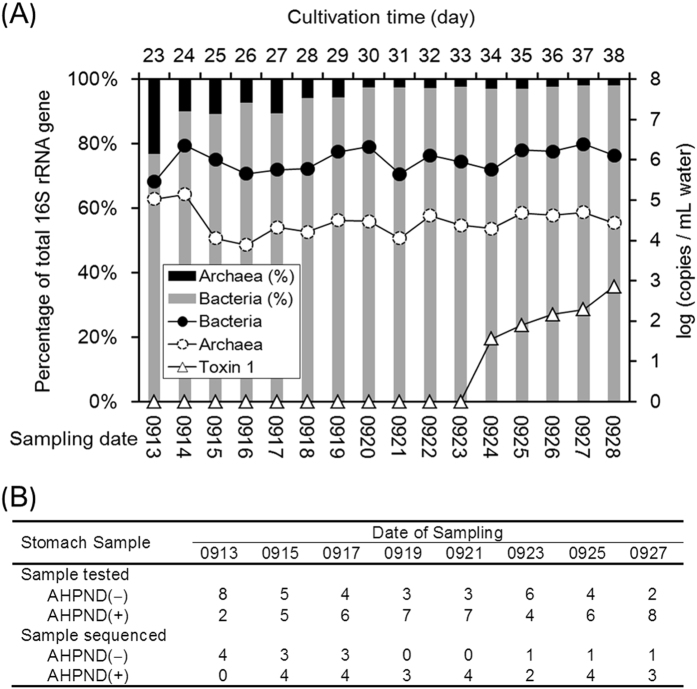
(**A**) Quantitative profiles of 16S rRNA genes of aquatic bacterial and archaeal populations and PirAB^vp^ gene (Toxin 1) in the shrimp cultivation pond. Histograms represent the relative abundances of *Bacteria* and *Archaea* in pond seawater samples at individual sampling points. (**B**) Numbers of stomach samples with (AHPND(+)) and without (AHPND(−)) PirAB^vp^ gene (Toxin 1) tested in this study. The samples with sufficient 16S rRNA gene amplicons obtained were then sequenced using the Miseq platform.

The daily monitoring data demonstrated that pH (7.4−7.7), alkalinity (79−102 mg/L), ammonia (<0.1 mg/L) and sulphate (79−163 mg/L) levels were relatively stable in the sampling period from Day 23 (Sep 13) to Day 38 (Sep 28) (Fig. S1). The concentrations of dissolved organic carbon (DOC) (2.0−5.9 mg/L) and nitrite (~3.0 mg/L) gradually increased, suggesting that eutrophication occurred in the pond seawater. Figure 1(A) presents the concentration variations in 16S rRNA and Toxin 1 genes (PirAB^vp^ gene) of free-living microorganisms analysed using quantitative PCR (qPCR). The results revealed that the concentrations of bacterial 16S rRNA genes were in the range of 10^5.5^–10^6.4^ copies/mL, which was higher than that of the archaeal counterparts (10^3.9^−10^5.1^ copies/mL). The proportion of bacterial to archaeal 16S rRNA gene quantity increased gradually from 76.8% on Day 23 to 97–98% after Day 30. The Toxin 1 gene was detected on Day 34 (Sep 24) at a concentration of 10^1.6^ copies/mL and increased to 10^2.9^ copies/mL within 4 days, indicating an AHPND outbreak in the shrimp farming pond; however, the ratio of Toxin 1 to bacterial 16S rRNA copies was relatively low (0.01–0.06%). Because the pale hepatopancreas of shrimp (typical syndrome of AHPND), and numerous dead shrimp on Day 37 were observed, the shrimp were thus harvested (survival rate, 94%) (Table S1).

### Screening of healthy and diseased shrimp

In this study, the AHPND plasmid and Toxin 1 gene in stomach samples of 10 individuals from each sampling day were detected using the IQ REAL real-time PCR. The results suggested that the AHPND plasmid was prevalent in nearly all (94.6%) shrimp samples. As shown in Fig. 1(B), the Toxin 1 gene was first detected in the stomach samples collected on Day 23 (Sep 13), at least 11 days before it could be detected in the aquatic samples. Due to insufficient amounts of 16S rRNA gene amplicons obtained, only 13 and 24 shrimp that exhibited negative (samples 1–13) and positive (samples 14–37) signals for the Toxin 1 gene (Table S2), herein defined as AHPND(−) and AHPND(+) samples, respectively, were subjected to a high-throughput sequencing analysis.

### Temporal variation of bacterial diversity

**Figure 2:**
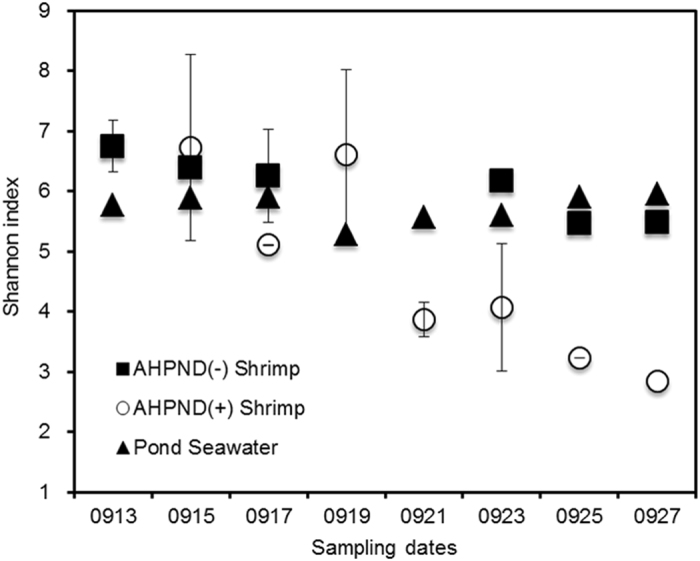
Distribution of Shannon diversity index values for samples of AHPND(−) shrimp, AHPND(+) shrimp, and pond seawater during shrimp cultivation.

The prokaryotic populations in the shrimp stomach and pond seawater samples on every alternate sampling day were analysed using tag-encoded high-throughput sequencing of the V3–V4 region of 16S rRNA gene amplicons. The resulting data revealed that more than 99.1% of the total quality reads were assigned to *Bacteria*; in addition, most stomach samples could not be successfully amplified with *Archaea*-specific primers. This result suggested that bacterial members were far more predominant than the archaeal members in the shrimp stomachs. In this study, a random subsampling of read data sets was performed to ensure an even sampling depth (14, 000 reads per library) with an average length of 412 bp for each sample (Table S3). The richness index (Chao 1) was estimated to be 525, 698 and 994 on average per samples from AHPND(−), and AHPND(+) stomach and pond seawater, respectively, suggesting that pond seawater harboured significantly higher richness of bacterial species than the shrimp stomachs by approximately 1.4−1.9 times (Tukey test, *P* < 0.00001). Figure 2 presents the distribution of Shannon index values and shows that the degree of complexity of bacterial diversity in pond seawater was relatively stable (5.73 ± 0.23), whereas that in AHPND(−) shrimp slightly declined from 6.75 ± 0.50 to 5.48. In AHPND(+) shrimp, the bacterial diversity was largely simplified because the Shannon index declined from 6.72 ± 0.37 to 2.85 ± 0.74 on average. However, the Tukey pairwise test indicated that the differences between the AHPND(+) samples and AHPND(−) and pond seawater samples were not significant (*P* > 0.3156). Notably, a tipping point occurred around Sep 19–20 when the degree of bacterial diversity of the AHPND(+) samples suddenly shifted. The results suggested that the AHPND infection markedly altered the bacterial diversity in the shrimp stomachs.

### Principal co-ordinates analysis

**Figure 3:**
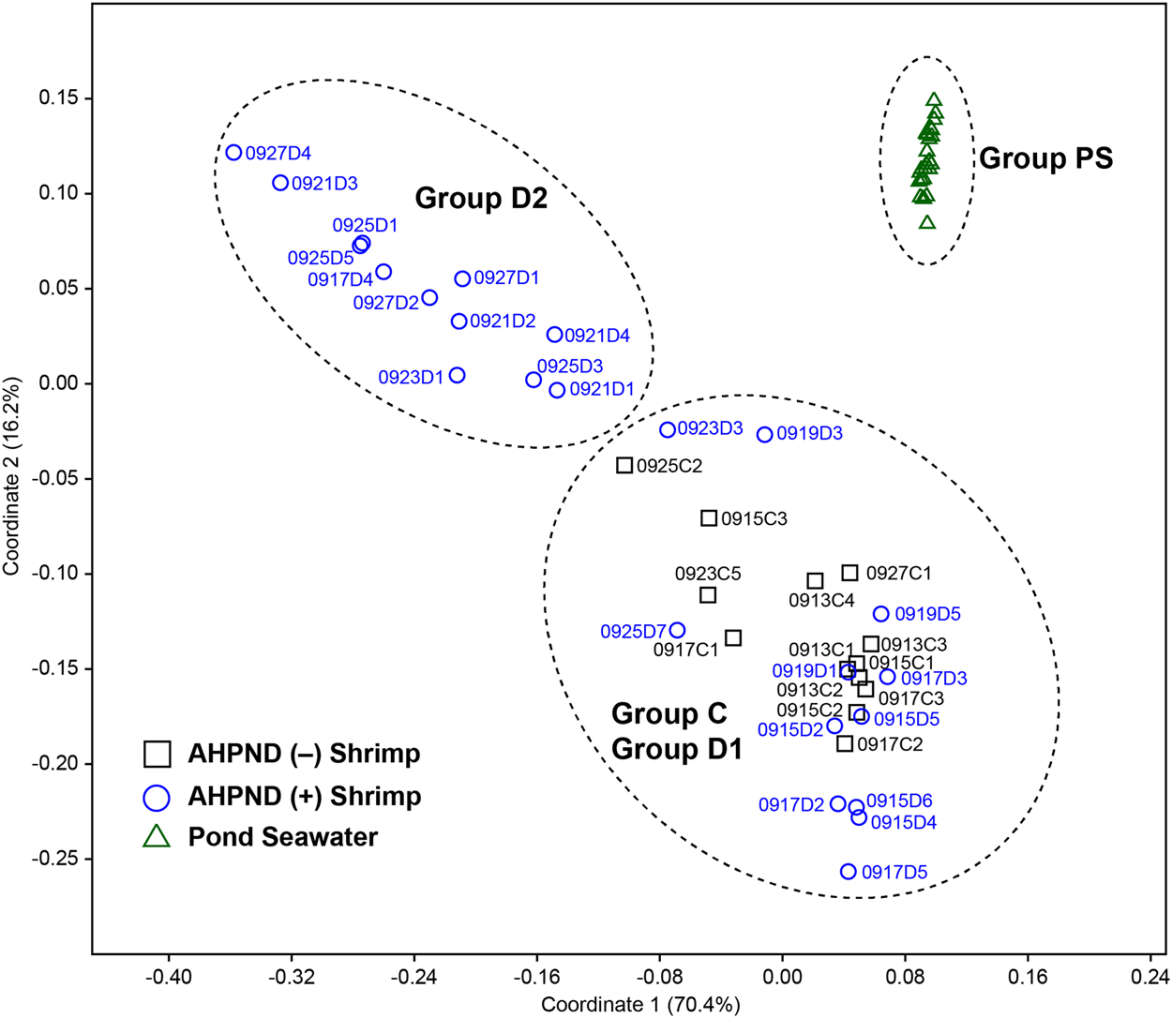
Principal co-ordinates analysis of bacterial community structures of the samples collected from the pond seawater (Group PS), healthy shrimp stomach (Group C), and shrimp stomach affected by AHPND (Groups D1 and D2).

The results of a principal co-ordinates analysis (PCoA) revealed that the 62 samples analysed in this study could be classified into three distinct clusters (ANOSIM analysis, *P* < 0.0001), indicating the degree of microbiome similarity among the sample types (Fig. 3). Cluster I comprised only pond seawater samples and were distinctly separated from the bacterial communities of shrimp samples. The shrimp samples varied widely in the coordination space and were grouped to two other clusters. Cluster II consisted of all samples collected from AHPND(−) shrimp and several AHPND(+) samples (designated as Group D1). Because most Group D1 samples were obtained before Sep 19 (except for samples 0923D3 and 0925D7), they were likely in the early stage of AHPND development, which did not pose evident effects on the microbiome yet. Thus, AHPND(+) samples shared a similar community structure with AHPND(−) samples. Cluster III represented only AHPND(+) samples (designated as Group D2), most of which were collected from Sep 21 to Sep 27. Consequently, the Group D2 samples were similar to the microbiome severely affected by AHPND. The overall results suggested that the microbiomes were discriminable according to their environments (pond seawater vs. shrimp stomachs) and AHPND infection status (healthy/early stage vs. late stage).

### Dynamics of core microbiomes

**Figure 4:**
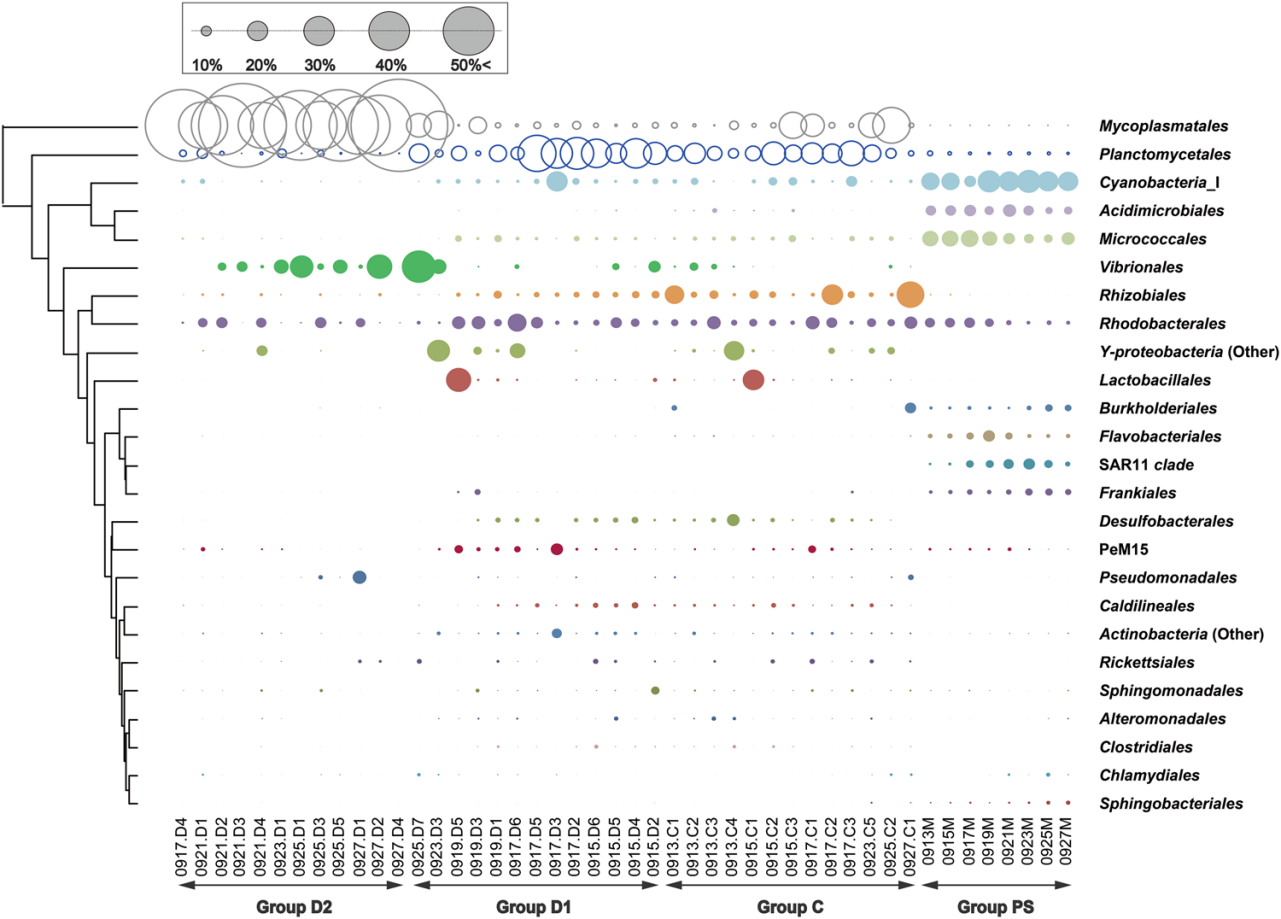
Distribution of sequence read abundance of core bacterial populations (sample occupancy > 85%) at the order level in each sample of pond seawater (Group PS), healthy shrimp stomach (Group C), and shrimp stomach affected by AHPND (Groups D1 and D2). The bacterial taxa and their clustering dendrograms are displayed on the right and left sides of the figure, respectively. Only the taxa with sequence read abundance > 4% in the samples are shown. Bubble size represents the relative abundance.

Approximately 96.9% of the total reads were distributed into 54–88 order taxa with a single taxon read abundance of >0.1% in each library. These reads were assigned to 26, 30, and 11 bacterial order taxa in 83–85% of pond seawater, AHPND(−), and AHPND(+) samples respectively, and were thus considered as members of core microbiomes on each sample type, in accordance with the core-satellite model^22^. Figure 4 shows the distribution of the core taxa with read percentages > 4%. The abundance data of taxa in pond seawater samples were relatively consistent among the triplicate analyses (CV = 0.24% ± 0.24%). Notably, high-magnitude differences in sequence abundance were observed in several taxa in the shrimp samples collected on the same day, suggesting the inter-variations of individual stomach microbiomes. For pond seawater samples, *Cyanobacteria* (subsection I), *Micrococcales*, and *Acidimicrobiales* were among the top three abundant order taxa detected. Their profiles remained stable over the course of AHPND outbreak. The profiles of medium-abundant populations from *Rhodobacterales*, *Burkholderiales*, *Flavobacteriales*, SAR11 clade, *Frankiales*, *Planctomycetales*, and PeM15 exhibited notable variations. Actually, the microbiome of pond seawater was subjected to dynamic progression (Fig. S2). The members of *Alphaproteobacteria* (*Rhizobiales* and *Rhodobacterales*), *Planctomycetales*, *Mycoplasmatales*, *Micrococcales*, *Cyanobacteria* (subsection I), and PeM15 represented the abundant populations in the AHPND(−) and Group D1 samples. The abundance of these populations, except for *Mycoplasmatales*, drastically declined in the AHPND(+) samples. The structure of core microbiomes of shrimp stomachs were highly altered in the Group D2 samples because the community shifted to be highly dominated by *Mycoplasmatales* and *Vibrionales* populations.

### Preferential habitats of OTUs

**Figure 5:**
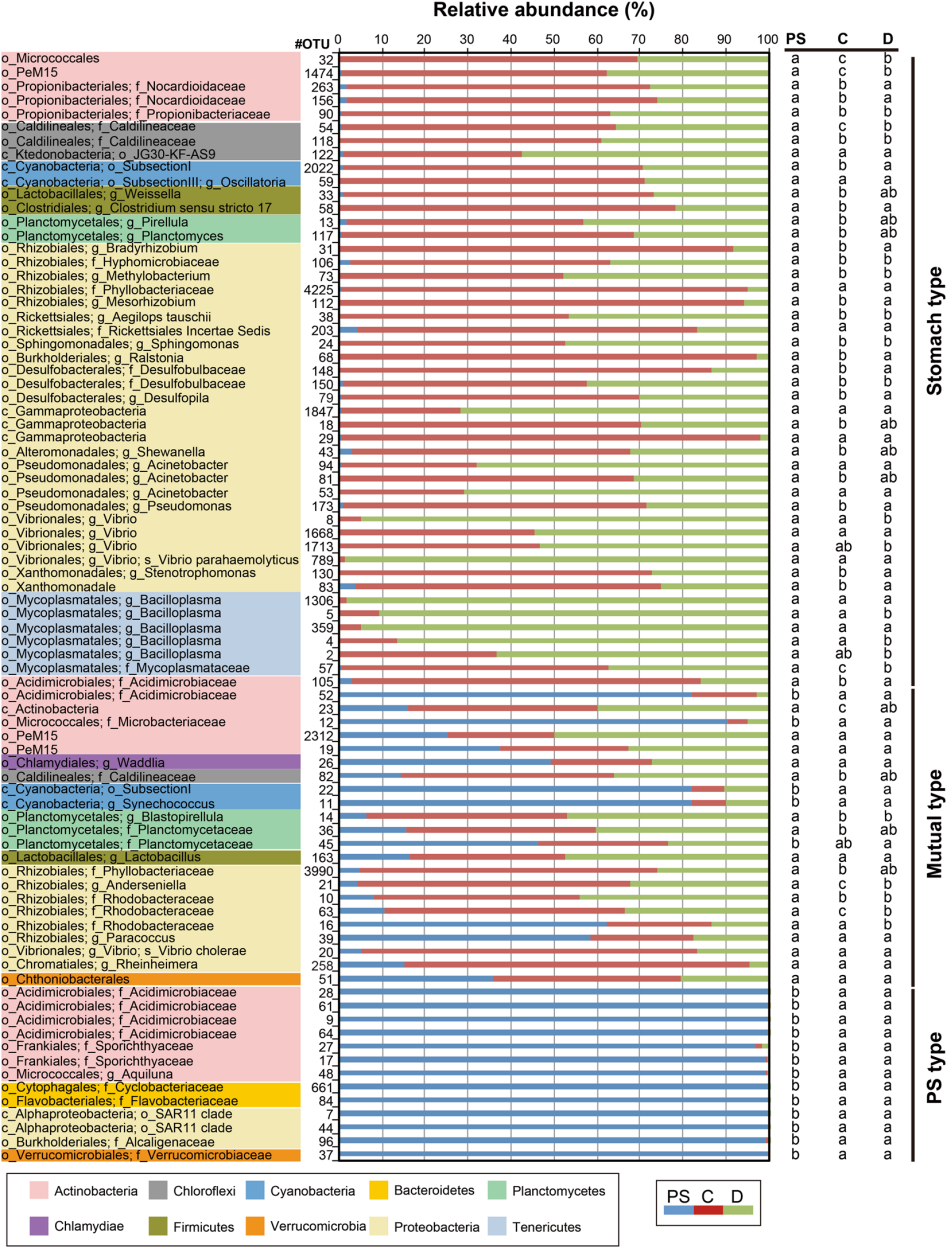
Relative abundance of abundant bacterial populations (82 OTUs) distributed in the samples of pond seawater (PS), healthy shrimp stomach (C), and AHPND-affected shrimp stomach (D). The results of Tukey multiple-comparison test of preferential existence on each OTU in the pond seawater (PS type), stomach (S type), and both environments (M type) are shown on the right side of the figure with different letters to indicate significantly different values (*P* < 0.05). The phylogeny of each OTU was coloured and illustrated on the left side of the figure.

A total of 82 core OTUs with averaged abundance > 0.1% in each sample type were further characterized for their preferable habitats. A total of 13 OTUs were detected exclusively or with high relative abundance (>95%) in pond seawater samples and were thus designated as the PS-type (pond seawater type) species (Fig. 5). These were phylogenetically related to *Verrucomicrobia*, *Proteobacteria*, *Bacteroidetes*, and *Actinobacteria* and preferably inhabit pond seawater in higher relative abundance than the environment in stomach, suggesting that their growth was affected by water quality. Similarly, 30 OTUs were defined as the S-type (stomach type) species in accordance with their significantly higher relative abundance in group C shrimp stomachs than in pond seawater (Tukey test, *P* < 0.05). The number decreased to 16 with the group D samples, suggesting the effects of AHPND on the population decolonization from the stomach. Another 17 OTUs also had higher relative abundance in group C shrimp stomachs than in pond seawater but their *P*-values did not reach a statistically significant level (Tukey test, *P* > 0.05). Notably, several OTUs related to *Candidatus* Bacilloplasma (OTUs, 4, 5, 359, and 1306) and *Vibrio* (OTUs 8 and 789) were highly associated with the AHPND(+) shrimp (Tukey test, *P* = 0.02-0.001 for OTUs 4, 5 and 8). The remaining 22 OTUs were the M-type (mutual type) species, which were abundant in both seawater and stomach environments, and thus had crosstalk on the microbiomes of different environments.

### Bacterial interaction networks

To evaluate the effects of AHPND infection on the species-to-species interactions within stomachs, bacterial network analyses were performed with Metagenomic Microbial Interaction Simulator (MetaMIS) for AHPND(−) and AHPND(+) samples, respectively. The MetaMIS is a Lotka–Volterra model-based tool for evaluating microbial interactions^23^. By using an abundance-ranking strategy, this tool systematically examines interaction patterns such as mutualism (+/+), competition (−/−), parasitism or predation (+/−), commensalism (+/0), and amensalism (−/0). The results revealed a high connectivity among the core OTUs within the stomach microbiome (Fig. S3). Among 138 OTUs analysed, 29 and 25 (in all, 36 nonrepeating OTUs) served as the hubs in the networks of AHPND(−) and AHPND(+) samples, respectively. These populations are taxonomically diversified and are distributed within 11 order taxa, including *Ahphaproteobacteria* (*Anderseniella*, *Bradyrhizobium*), *Betaproteobacteria* (*Ralstonia*), *Gammaproteobacteria* (*Vibrio, Shewanella*, and *Acinetobacter*), *Mycoplasmatales* (*Bacilloplasma*), *Cyanobacteria* (*Synechococcus*), *Plancyomycetales* (*Pirellula* and *Blastopirellula*), *Actinobacteria*, *Lactobacillales* (*Weissella*), *Caldilineales*, *Chlamydiales*, and *Clostridiales*. Notably, approximately 10% of the hub OTUs were commonly present at low abundance (<1%), suggesting that even at low abundance, the OTUs played a key role in modulating the inter-bacterial interactions in a stomach microbial consortium. In both networks, the numbers of shared hub OTUs were higher than those of unique OTUs [Fig. S3(a,b)]. A set of 18 hub OTUs were shared by all shrimp samples, whereas 11 and 7 OTUs were specific to the AHPND(−) and AHPND(+) microbiomes, respectively [Fig. S3(c)]. Although most hub populations belonged to the S-type, eight M-type OTUs (OTUs 11, 22, 23, 36, 26, 63, 12, and 19) were detected, suggesting that the factors of host and surrounding environments could affect the bacterial networks in the shrimp stomachs. The key hubs in the networks appeared to be OTU dependent. For example, various hub OTUs belonging to the same *Vibrio* genus were detected specifically in the AHPND(−) (OTU20) and AHPND(+) (OTUs 8, 1668, and 1713) microbiomes, respectively.

**Figure 6:**
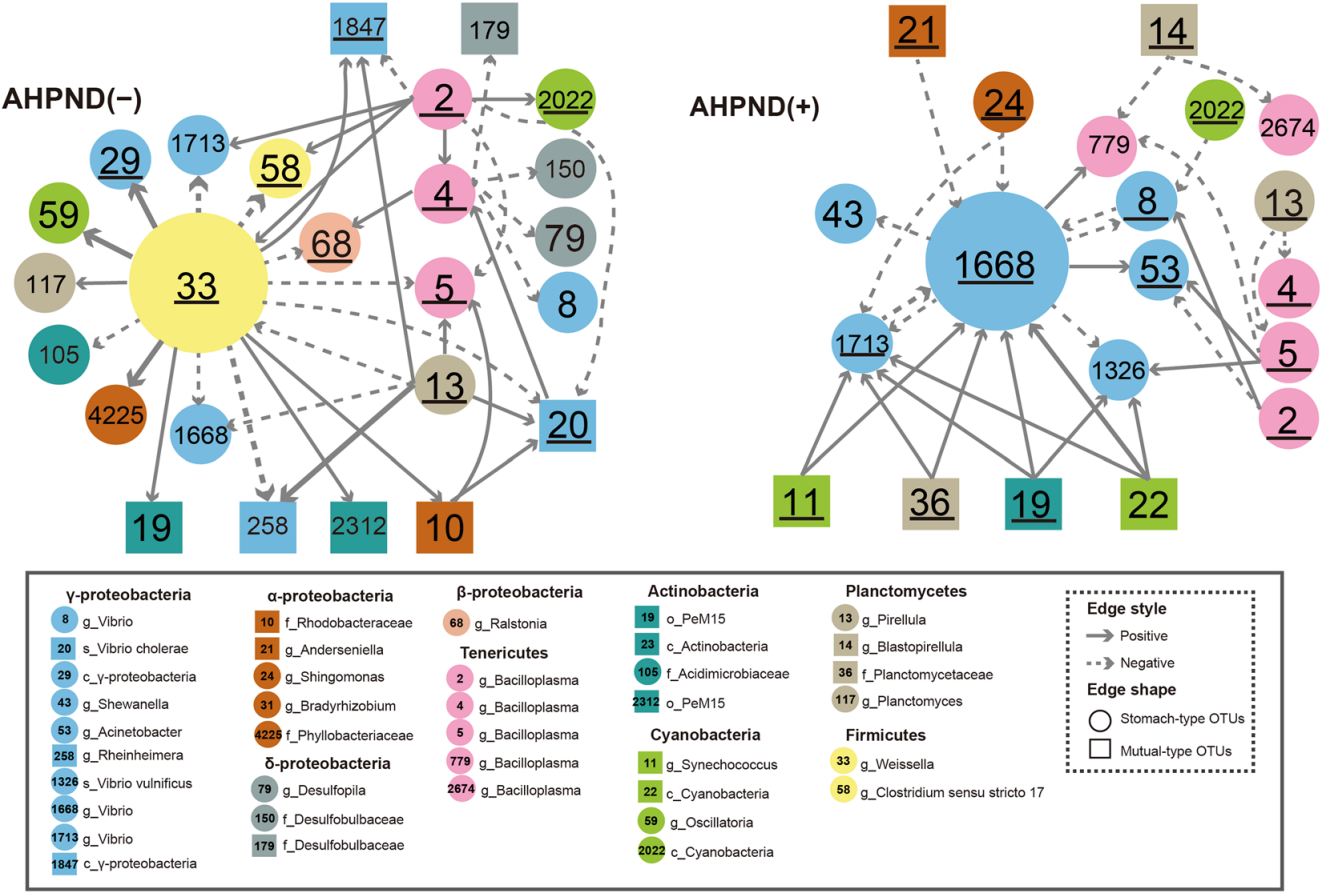
*Vibrio*-related consensus interactions in the AHPND(−) and AHPND(+) stomach communities. The interaction strength was ranked among the top 1% of the entire network. The solid (or dashed) arrow represents the activation (or repression). The hub OTUs are underlined.

Because of the role of *Vibrio* species in hosting the AHPND plasmid, we particularly studied the bacterial interactions in these species. Figure 6 presents the detailed interactions between the *Vibrio* OTUs and the co-occurring OTUs, which were ranked among the top 1% interaction strength (>±6.0) in the consensus networks. The network analysis suggested that the abundance of *Vibrio* OTUs in the shrimp stomachs tended to be greatly modulated by both S-type and M-type bacterial populations. As predicted, the *Weissella* OTU33 of *Lactobacillales* was an important hub and connected most arrows [commensalism (+/0) or amensalism (−/0)] to the populations in the AHPND(−) network (top 1% strength). Because amensalism (−/0) interaction was displayed, the *Weissella* OTU33 might inhibit *Vibrio* OTUs. In contrast to only one *Vibrio* OTU as the hub in the AHPND(−) network, the roles of three *Vibrio* OTUs (1668, 1713, and 8) in the AHPND(+) network were highlighted. In particular, OTU1668 received commensalism (+/0) arrows from several M-type OTUs, suggesting an influence of water quality on vibrios. In addition to the competition (−/−) effects with OTU1668, the abundance of OTU8 might be stimulated by S-type OTU2 (*Candidatus* Bacilloplasma), but suppressed by the S-type cyanobacterial OTU2022. The cross-environment species with their abundance varying with water quality could have a marked effect on *Vibrio* OTU8 in the co-occurrence of OTU1668.

## Discussion

We successfully tracked the diversity and dynamics of bacterial populations in shrimp stomachs and pond seawater during an outbreak of AHPND in a field grow-out pond. The present study is the first to report the progressive alternation of stomach microbiome caused by AHPND in shrimp. The results further suggested a marked shift in bacterial composition, and interaction network in the AHPND(+) shrimp stomachs compared with the healthy counterpart. The strong interactions of the crucial *Vibrio* populations with S- and M-type populations revealed that both host physiological conditions and eutrophication of water quality might contribute to the development of AHPND(+) microbiomes.

The bacterial community structure and dynamics in pond seawater were distinctly different from those of shrimp stomachs in the sampling period (Fig. 3), which suggests the varied development process of microbiomes in different environments. A low similarity (13–35%) of bacterial communities between seawater and intestine in shrimp was observed in previous studies^24, 25^. Certainly, the pond seawater was in a higher oxidizing condition because of the continuous oxygenation from the mechanical aeration and microbial photosynthesis. The shrimp stomachs harboured populations of several anaerobic bacterial taxa (such as *Desulfobacterales* and *Caldilineales*) with far higher abundance than seawater (Fig. 4), implying a prevalence of reducing conditions. The occurrence and abundance of S-type microorganisms in the intestine may be largely dependent on the host phylogeny^21^ and physiological factors^26^, and the intestinal microbiome could greatly vary at different growth stages of the shrimp lifecycle^16^. In addition, because a fraction of marine bacterial species could inhabit the stomach environment (Fig. 5), the M-type populations affected the stomach microbiomes in association with surrounding environmental factors. The gradual shift in the microbiome of shrimp stomach could be also attributed to the transit bacterial population due to a change of the feeding conditions with the infected shrimp^27^. The findings could be supported by the studies of fish gut communities^28, 29^, suggesting that after stocking for 23 days, the deterministic process (host selection) have exerted more effects on stomach assemblages of the shrimp juvenile than the stochastic process (random dispersal)^26^. Our results further revealed that when shrimp was diseased with AHPND, the ability to select bacterial residents in stomach markedly decreased, thus the stochastic process that is associated with environmental factors becoming more important to shape the stomach microbiota. The progressive succession of microbiota from health to disease status is most probably a universal response of digestive tract microbiota to disease, which is concordant in both shrimp stomach and gut^27^.

Recently, AHPND in shrimp has been reported to be caused by the pore-forming toxins encoded by PirAB^vp^ genes in the AHPND plasmid hosted by *V*. *parahaemolyticus* ^6^. In this research, all the corresponding agents (toxin gene, plasmid, and *V*. *parahaemolyticus*) were detected in the AHPND(+) samples, as well as the observation of pale hepatopancreas of shrimp, thus confirming the AHPND infection. We also inspected the levels of white spot syndrome virus for all shrimp samples tested in this study, but the resulting data was far lower (<10^−5^ copies/host genome copy) than the level (10^−2^ copies/host genome copy) to induce a white spot disease. Consistent with previous studies^6, 14, 30^, in the present study, the presence of bacterial host and AHPND plasmid without PirAB^vp^ did not provide an evident assessment for AHPND infection in shrimp. Our data suggested that AHPND infection is more precisely associated with the presence of the Toxin 1 gene (PirAB^vp^), and the shrimp stomach samples were more sensitive to the analysis of Toxin 1 gene than that of water samples (Fig. 1). The consistency of results obtained using qPCR analysis of the Toxin 1 gene and PCoA of microbiomes suggested the effectiveness of stomach microbiome as an indicator for assessing the status of shrimp health^10, 31, 32^. The PCoA results of this study further revealed two distinct stages of the microbiome assembly with AHPND. The initial AHPND infection from Sep 15 to Sep 19 did not immediately lead to a significant change in the overall structure of the bacterial communities in the shrimp stomachs (Figs 3 and 4). From Sep 21 to Sep 27, reduced feeding, pale hepatopancreas and dead shrimp were observed, and the effects of AHPND could have been occurring during this time, which was reflected by a ready loss in bacterial diversity and consequently, a great shift in the stomach microbiome. Because the rapid development of the AHPND-affected microbiome coincided with the occurrence of heavy rain on Sep 19, the temporary salinity stress that may have caused an immune change in shrimp^33, 34^ was thus speculated to have opened a niche for detrimental colonizers such as the AHPND agent *V*. *parahaemolyticus*. No significant difference (T-test, *P* = 0.4719) in the copies of the Toxin 1 gene was observed in the AHPND(+) samples at both stages (Table S2). This result was consistent with that of a recent study, revealing that the virulence of *Pir* ^*vp*^ toxin had no correlation with the gene copies^35^. To precisely determine the status of AHPND infection in shrimp, a quantitative analysis of the Toxin 1 gene in combination with microbiome analysis should be conducted.

According to a previous study^26^ and the present study, populations of *Alphaproteobacteria* (*Rhodobacterales* and *Rhizobiales*) and *Planctomycetales* dominated the intestinal systems of healthy shrimp. The dominance had been attributed to the effects of host selection that was more important than that of the stochastic process in the healthy shrimp adults^27^. However, in diseased shrimp, these populations became minor, and the populations of *Vibrionales*/*Gammaproteobacteria* usually became predominant. During AHPND infection, remarkably abundant *Mycoplasmatales* were observed in this study. The members of *Mycoplasmatales* and *Vibrionales* are actually common inhabitants of the digestive system of several shrimp species^16, 18, 19, 24, 36^. Because the two populations were crucial in the Group D2 samples (Fig. 4), we investigated them in additional detail.

The sequence analysis detected 21 out of 27 *Mycoplasmatales*-related OTUs in affiliation with *Candidatus* Bacilloplasma, a genus taxon of symbiotic populations with no isolated strain available yet^37^. OTUs 2, 4, and 5 were numerically abundant (up to 86.8%) in the shrimp samples in this study. As detailed in the phylogenetic tree, the *Candidatus* Bacilloplasma OTUs were clustered into several subgroups in which the neighbouring sequences were obtained from sea animals, such as shrimp^16, 38^, crab^39^, catfish^40^, lobster^41^, and turbot^42^ (Fig. S4). The findings suggested that this yet-to-be-cultured population was highly diversified and played an important role in the gut microbiome of sea creatures. From *Vibrionales*, six OTUs were classified into the genus *Vibrio*, of which the detected sequences of OTU20, OTU1326, and OTU1668 were identical to those of *Vibrio cholera*, *Vibrio vulnificus*, and *Vibrio fluvialis*, respectively. They were typically detected in the AHPND(−) and Group D1 samples (Fig. S5). Distinguishing the non-pathogenic *Vibrio* species from the pathogenic ones by using the 16S rRNA gene as a marker is challenging. Although several isolates in the *Harveyi* clade were reported to cause AHPND, they could differ in virulence^6^. However, because of identical sequences of the AHPND-related pathogenic strains VP-3HP and VP-5HP^43^, the *Vibrio* OTU8 was suspected to be the causative microorganism. Certainly, the dominance of the *Vibrio* OTU8 in the stomach was observed with D2 samples after salinity stress (Fig. S5). The species was found in high abundance (8.28% ± 10.92%) in the Group D2 samples, but usually in low abundance in the non-AHPND shrimp (0.29% ± 0.85%) and seawater (0.01% ± 0.01%) (T-test, *P* = 0.019-0.029). Similarly, OTU789 was identified as a member of *V*. *parahaemolyticus*; however, its abundance (0.14% ± 0.59%) was much lower than that of OTU8 in the diseased shrimp. Whether the AHPND was caused by more than one strain in this study remains unclear because a previous study reported that several AHPND-causing *V*. *parahaemolyticus* strains could be obtained from a single cultivation pond^43^.

As predicted by MetaMIS, several OTUs of *Vibrio* and *Candidatus* Bacilloplasma constituted the core hubs and could interact with each other in the microbiome networks of the shrimp stomachs (Figs S3 and 6). The abundance of *Vibrio* OTU8 was likely stimulated by *Candidatus* Bacilloplasma OTU2 in the AHPND(+) microbiome, but suppressed by *Candidatus* Bacilloplasma OTU4 in the AHPND(−) microbiome. This prediction can be coincided by a very recent finding that the AHPND would effect upon the colonization of *V*. *parahaemolyticus* on stomach linings^44^. Because *Candidatus* Bacilloplasma is commensal and is attached to the gastrointestinal tract of crustaceans^37^, it would have stronger interactions with *Vibrio* OTU8 for enhancement or inhibition. Notably, different *Candidatus* Bacilloplasma OTUs interacted with *Vibrio* OTU8 in the networks, suggesting that the population likely processed the subspecies diversity, and varied subspecies were involved in the observed commensalism in the microbiome. Thus far, information on this yet-to-be-cultured *Candidatus* Bacilloplasma population is relatively limited, and additional studies are warranted.

The effects of AHPND disturbed the balance of the bacterial networks in the stomachs and shifted the bacterial co-occurrence pattern. The MetaMIS analysis results suggested that *Weissella* OTU33 and *Vibrio* OTU1668 served as the major hubs in the microbiomes of the shrimp stomachs (Fig. 6). The *Weissella* and some *Vibrio* species are facultatively anaerobic populations^45, 46^. Several strains of *Weissella* and *Vibrio* have been reported to be probiotic and reduce the infectivity and persistence of digestive pathogens^47–49^. Unlike an evenly distributed abundance (0.97–1.24%) of *Vibrio* OTU1668 in healthy and diseased shrimp, the abundance of *Weissella* OTU33 was higher in the AHPND(−) samples (2.37% ± 5.35%) than in the Group D1 (1.60% ± 2.86%) (T-test, *P* = 0.3336) and Group D2 (0.06% ± 0.06%) samples (T-test, *P* = 0.083), suggesting that *Weissella* OTU33 is a potential indicator for the health status of the stomach microbiomes in white shrimp.

Because eutrophication occurred with the increasing nutrient loads, cyanobacterial populations became prominent in the shrimp pond (Fig. 4). The dominance of cyanobacteria populations was reported to be associated with several environmental factors such as light, salinity, temperature, and nutrient levels, and it could regulate the water quality in the pond seawater^50^. Since cyanobacterial cells were not a preferential ingestion diet for shrimp^51^, the population in the stomachs may play an ecological role. As shown in Fig. 4, the lower abundance of cyanobacteria in Group D2 shrimp relative to those in the AHPND(−) and Group D1 shrimp may indicate the loss of the redox regulation capability in shrimp severely infected with AHPND. Despite the low abundance, the MetaMIS analysis suggested that two M-type OTUs (OTU11 and OTU22) and one S-type (OTU2022) served as the core hubs with strong interactions with *Vibrio* spp. in the consensus bacterial networks of the AHPND(+) shrimp stomachs (Fig. 6). Actually, these cyanobacteria were closely related to the *Synechococcus*, which have been reported to be dominant in the coastal seawater or brackish water^52, 53^, and potentially exert toxic effects on marine invertebrates^54^. Whether the three *Synechococcus* OTUs can produce toxins in the shrimp stomach could not be validated with the 16S amplicon sequencing approach, even though our current study used the Illumina sequencing technology to resolve the bacterial groups at the species or OTU level. With a better understanding of microbiota in shrimp and surrounding environment, future studies would aim to elaborate their functions through multiple functional meta-omics approach. Such efforts would be crucial for assessing the association of bacterial species already known and the shrimp health, as well as the ecological roles of the uncultured group. For example, the PeM15 populations appeared to play an important role in shrimp stomach, but their functions remains unclear.

In summary, the present study carried out 16S rRNA-based Illumina sequencing on microbiota sampled from healthy and AHPND-affected shrimp stomachs, and pond seawater, and provided new insights into the dynamics of microbiomes in shrimp stomachs and pond seawater. The results suggested that the occurrence of AHPND in shrimp could be a consequence of changes in stomach bacterial communities with disruptions in species-to-species co-occurrence patterns, which could be useful for developing microbial early warning systems and probiotic products for shrimp pond farming for more efficient management of shrimp farming ponds and for minimizing the outbreak of AHPND.

## Methods

### Shrimp farming

Shrimp cultivation was initiated with a stocking density of approximately 79 postlarvae/m^2^. Approximately 500,000 white shrimp (*Litopenaeus vannamei*) larvae were stocked in a rectangular pond with an area of 6300 m^2^ and water depth of 1.5 m in Ben Tre Province, Vietnam, on Aug 20, 2015. The specific pathogen-free (SPF) larvae (PL20) were validated to be free of infectious agents, including the World Organisation for Animal Health (OIE)-listed shrimp pathogens (white spot syndrome virus, infectious hypodermal and haematopoietic necrosis virus, yellow head virus, Taura syndrome virus, and infectious myonecrosis virus) and AHPND-causing *V*. *parahaemolyticus*. Before stocking, the seawater was sterilized with dilute hypochlorous acid. The pond sediment was treated with persulfate for several days and then exposed to sunlight for weeks. The larvae were fed four times a day. Because rainfall was frequent in the area, minerals such as dolomite, silicate (Khoang), and calcium oxide were supplied to maintain levels of alkalinity, mineral nutrients, and salinity (2–2.2%). Notably, extraordinarily heavy rain occurred on the 29th stocking day (Sep 19). The details of the shrimp cultivation in this study are listed in Table S1.

### Sampling and sample pretreatment

Sampling was started from Day 23 (Sep 13) of pond rearing until Day 38 (Sep 28), one day before an urgent harvest. Pond seawater of approximately 45 cm in depth from three locations at least 3 m away from the pond border was collected during the daytime. The suspended microbial cells were recovered by filtering 200–250 mL of water samples through a 0.2-μm sterilized membrane and preserved at −20 °C. Parameters of water quality were monitored daily (Fig. S1). For shrimp sampling, a total of ten shrimp were collected on each sampling day and placed in an RNA keeper solution (Protech Technology Enterprise Co., Ltd., Taiwan) and stocked at −20 °C until use. The stomach of each shrimp was collected and sliced into two parts: the small part (one fifth of the stomach) was used for AHPND detection, and the large part (four fifths of the stomach) was used for microbiome analysis.

### DNA recovery

The DNA of microbial cells in the membrane, small fractions, and large fractions of stomach samples were recovered using a bead-beating-based PowerWater DNA Isolation Kit (MoBio, USA), a DTAB/CTAB DNA extraction kit (Gene Reach Biotechnology Corps), and a DNeasy blood and tissue kit (Qiagen) according to the manufacturers’ protocols, respectively. The quality of the obtained genomic DNA was verified using a NanoVue spectrophotometer (GE, USA), and the concentrations were determined using the Quant-iT PicoGreen dsDNA Assay Kit (Invitrogen, USA). The DNA samples were stored at −20 °C until use for analysis.

### Analysis of AHPND-related markers with shrimp stomach samples

The stomach samples (fraction of small pieces) were analysed for AHPND-related markers by using TaqMan real-time PCR assays. Copies of the PirAB^vp^ gene (Toxin 1) and AHPND plasmid as well as the host genome in a stomach sample were quantitatively determined using an IQ REAL™ AHPND/EMS Quantitative System and an IQ REAL™ WSSV Quantitative System (Gene Reach Biotechnology Corps) on a CFX96 real-time system (Bio-Rad, USA) according to the suppliers’ instructions. In brief, each TaqMan qPCR reaction mixture (25 µL) contained 1X of Real-Time PreMix, 4U of IQzyme DNA polymerase, and 2 μL of gDNA template. A two-step thermal condition was applied: 40 cycles of 93 °C for 15 s and 60 °C for 1 min. Artificial DNA-containing partial sequences of the PirAB^vp^ gene (Toxin 1) and AHPND-associated plasmid were used to construct the calibration curves.

### Quantitation of 16S rRNA genes

The concentrations of prokaryotic populations in pond seawater were assessed by determining the copies of 16S rRNA genes using the SYBR Green qPCR method. The qPCR experiments were performed on a CFX96 real-time PCR detection system (BioRad, USA) with *Bacteria*-specific and *Archaea*-specific primers for quantitatively analysing bacterial and archaeal 16S rRNA genes, respectively (Table S4). Each reaction mixture (20 µL) contained 1X of SYBR Premix Ex *Taq* (Takara, Japan), 200 mM of each primer, and 5 ng of DNA template. The DNA templates used for constructing the calibration curves were clonal 16S rRNA gene fragments obtained in the laboratory. A melting analysis was performed to verify the specificity of each reaction. All the qPCR experiments were conducted in triplicate.

### Barcoded amplicon sequencing

The compositions of bacterial populations were analysed using high-throughput sequencing of 16S rRNA amplicons as previously described^55, 56^. The hypervariable V3–V4 region of the bacterial 16S rRNA gene was amplified using a barcoded fusion primer set (Table S4) with a thermal cycling program comprising an initial denaturation at 95 °C for 2 min, followed by 30–35 cycles of annealing starting at 65 °C (ending at 55 °C) for 15 s, and extension at 68 °C for 30 s. The amplicons in triplicate samples were pooled and purified using an AMPureXP PCR Purification Kit (Agencourt, Brea, CA, USA) and quantified using a Qubit dsDNA HS Assay Kit on a Qubit 2.0 Fluorometer (Invitrogen, Carlsbad, CA, USA). For sequencing library preparation, Illumina adapters were attached to the amplicons by using a Nextera XT DNA Library Preparation Kit. Purified libraries were used for cluster generation, and a 2 × 300 bp paired-end sequencing run was performed on an Illunina Miseq platform (Illumina, Inc., San Diego CA, USA). The sequencing data obtained in this study were submitted to the NCBI Sequence Read Archive under the study accession number SRP102384.

### Data processing

Raw sequence reads were sorted according to their respective barcodes into individual libraries. The sequences of primers, barcodes, and adaptors were trimmed. The sequence reads shorter than 300 bp, chimerical sequences, and those containing ambiguous nucleotides were filtered to obtain quality reads and then clustered for OTUs (3% sequence cutoff) with USEARCH^57^. Paired-end reads were merged using FLASH^58^. The OTU sequences were taxonomically assigned to the SILVA 16S rRNA gene database and taxonomy. After excluding the chloroplast-related sequences, rank abundance, species richness (Chao1), and species diversity (Shannon–Weiner index) indices were calculated using QIIME^59^. To normalize the uneven sequencing effects, the OTU table was 3X randomly rarefied subset of 14,000 sequences per sample. A weighted Unifrac-PCoA was performed to evaluate the structural similarities of the bacterial communities among the samples. A Tukey test and T-test were used to evaluate the differences of sample groups. Bacterial interaction network analysis was performed with the read abundance data at the OTU-level by using a stand-alone tool, MetaMIS^23^. The topology of the resulting bacterial network was visualised using Gephi.

## Electronic supplementary material

Supplementary Info

## References

[1] Hong XP, Lu LQ, Xu D (2016). Progress in research on acute hepatopancreatic necrosis disease (AHPND). Aquacult Int.

[2] Leaño, E. M. & Mohan, C. V. Early mortality syndrome threatens Asia’s shrimp farms. *Global Aquaculture Advocate*, 38-39 (2012).

[3] Lightner DV, Redman RM, Pantoja CR, Noble BL, Tran L (2012). Early mortality syndrome affects shrimp in Asia. Global Aquaculture Advocate.

[4] Tran L (2013). Determination of the infectious nature of the agent of acute hepatopancreatic necrosis syndrome affecting penaeid shrimp. Dis Aquat Organ.

[5] Flegel TW (2012). Historic emergence, impact and current status of shrimp pathogens in Asia. J. Invertebr. Pathol..

[6] Lee CT (2015). The opportunistic marine pathogen *Vibrio parahaemolyticus* becomes virulent by acquiring a plasmid that expresses a deadly toxin. Proc Natl Acad Sci USA.

[7] Zorriehzahra MJ, Banaederakhshan R (2015). Early mortality syndrome (EMS) as new emerging reat in shrimp industry. Adv Anim Vet Sci.

[8] Sassone-Corsi M, Raffatellu M (2015). No vacancy: how beneficial microbes cooperate with immunity to provide colonization resistance to pathogens. J. Immunol.

[9] Baumler AJ, Sperandio V (2016). Interactions between the microbiota and pathogenic bacteria in the gut. Nature.

[10] Xiong J (2015). Changes in intestinal bacterial communities are closely associated with shrimp disease severity. Appl Microbiol Biotechnol.

[11] Xiong JB, Dai WF, Li CH (2016). Advances, challenges, and directions in shrimp disease control: the guidelines from an ecological perspective. Appl Microbiol Biotechnol.

[12] De Schryver P, Defoirdt T, Sorgeloos P (2014). Early mortality syndrome outbreaks: a microbial management issue in shrimp farming?. PLoS Pathog.

[13] Johnson CN (2013). Fitness factors in vibrios: a mini-review. Microb Ecol.

[14] Lai HC (2015). Pathogenesis of acute hepatopancreatic necrosis disease (AHPND) in shrimp. Fish Shellfish Immunol.

[15] Xiong J (2014). The temporal scaling of bacterioplankton composition: high turnover and predictability during shrimp cultivation. Microb Ecol.

[16] Rungrassamee, W. *et al*. Bacterial population in intestines of the black tiger shrimp (*Penaeus monodon*) under different growth stages. *Plos One* **8**, doi:10.1371/journal.pone.0060802 (2013). 10.1371/journal.pone.0060802 PMC3618293 23577162

[17] Zhang H (2016). Dynamic changes of microbial communities in *Litopenaeus vannamei* cultures and the effects of environmental factors. Aquaculture.

[18] Zhang ML (2014). Characterization of the intestinal microbiota in Pacific white shrimp, *Litopenaeus vannamei*, fed diets with different lipid sources. Aquaculture.

[19] Chaiyapechara S (2012). Bacterial community associated with the intestinal tract of *P*. *monodon* in commercial farms. Microb Ecol.

[20] Rungrassamee, W. *et al*. Characterization of intestinal bacteria in wild and domesticated adult black tiger shrimp (*Penaeus monodon*). *Plos One* **9**, doi:10.1371/journal.pone.0091853 (2014). 10.1371/journal.pone.0091853 PMC3950284 24618668

[21] Tzeng, T. D. *et al*. Effects of host phylogeny and habitats on gut microbiomes of oriental river prawn (*Macrobrachium nipponense*). *Plos One* **10**, doi:10.1371/journal.pone.0132860 (2015). 10.1371/journal.pone.0132860 PMC4500556 26168244

[22] Hanski, I. Dynamics of regional distribution: the core and satellite species hypothesis. *Oikos*, 210-221 (1982).

[23] Shaw, G. T. W., Pao, Y. Y. & Wang, D. MetaMIS: a metagenomic microbial interaction simulator based on microbial community profiles. *Bmc Bioinformatics* **17**, doi:10.1186/s12859-016-1359-0 (2016). 10.1186/s12859-016-1359-0 PMC5124289 27887570

[24] Cardona, E. *et al*. Bacterial community characterization of water and intestine of the shrimp *Litopenaeus stylirostris* in a biofloc system. *Bmc Microbiol* **16**, doi:10.1186/s12866-016-0770-z (2016). 10.1186/s12866-016-0770-z PMC4952143 27435866

[25] Johnson CN, Barnes S, Ogle J, Grimes DJ (2008). Microbial community analysis of water, foregut, and hindgut during growth of pacific white shrimp, *Litopenaeus vannamei*, in closed-system aquaculture (vol 39, pg 251, 2008). J World Aquacult Soc.

[26] Zhu J (2016). Contrasting ecological processes and functional compositions between intestinal bacterial community in healthy and diseased shrimp. Microb Ecol.

[27] Xiong J (2017). Integrating gut microbiota immaturity and disease-discriminatory taxa to diagnose the initiation and severity of shrimp disease. Environ Microbiol.

[28] Sullam KE (2012). Environmental and ecological factors that shape the gut bacterial communities of fish: a meta‐analysis. Mol Ecol.

[29] Nayak SK (2010). Role of gastrointestinal microbiota in fish. Aquacult Res.

[30] Yang, Y. T. *et al*. Draft genome sequences of four strains of *Vibrio parahaemolyticus*, three of which cause early mortality syndrome/acute hepatopancreatic necrosis disease in shrimp in China and Thailand. *Genome Announc* **2**, doi:10.1128/genomeA.00816-14 (2014). 10.1128/genomeA.00816-14 PMC4155583 25189578

[31] Berry D, Reinisch W (2013). Intestinal microbiota: a source of novel biomarkers in inflammatory bowel diseases?. Best Pract Res Clin Gastroenterol.

[32] Clemente JC, Ursell LK, Parfrey LW, Knight R (2012). The impact of the gut microbiota on human health: an integrative view. Cell.

[33] Liao S (2012). Effect of nitrite on immunity of the white shrimp *Litopenaeus vannamei* at low temperture and low salinity. Ecotoxicology.

[34] Zhao Q, Pan L, Ren Q, Hu D (2015). Digital gene expression analysis in hemocytes of the white shrimp *Litopenaeus vannamei* in response to low salinity stress. Fish Shellfish Immunol.

[35] Tinwongger S (2016). Virulence of acute hepatopancreatic necrosis disease PirAB‐like relies on secreted proteins not on gene copy number. J Appl Microbiol.

[36] Liu H (2011). The intestinal microbial diversity in Chinese shrimp (*Fenneropenaeus chinensis*) as determined by PCR–DGGE and clone library analyses. Aquacult.

[37] Kostanjsek R, Strus J, Avgustin G (2007). “*Candidatus* Bacilloplasma,” a novel lineage of *Mollicutes* associated with the hindgut wall of the terrestrial isopod *Porcellio scaber* (Crustacea: Isopoda). Appl Environ Microbiol.

[38] Rungrassamee W, Klanchui A, Maibunkaew S, Karoonuthaisiri N (2016). Bacterial dynamics in intestines of the black tiger shrimp and the Pacific white shrimp during *Vibrio harveyi* exposure. J Invertebr Pathol.

[39] Chen, X. *et al*. Bacterial community associated with the intestinal tract of Chinese mitten crab (*Eriocheir sinensis*) farmed in Lake Tai, China. *PLoS One* **10**, e0123990, doi:10.1371/journal.pone.0123990 (2015). 10.1371/journal.pone.0123990 PMC4395229 25875449

[40] Wu S, Tian J, Wang G, Li W, Zou H (2012). Characterization of bacterial community in the stomach of yellow catfish (*Pelteobagrus fulvidraco*). World J Microbiol Biotechnol.

[41] Meziti A, Ramette A, Mente E, Kormas KA (2010). Temporal shifts of the Norway lobster (*Nephrops norvegicus*) gut bacterial communities. FEMS Microbiol Ecol.

[42] Xing M (2013). Taxonomic and functional metagenomic profiling of gastrointestinal tract microbiome of the farmed adult turbot (*Scophthalmus maximus*). FEMS Microbiol Ecol.

[43] Joshi J (2014). Variation in *Vibrio parahaemolyticus* isolates from a single Thai shrimp farm experiencing an outbreak of acute hepatopancreatic necrosis disease (AHPND). Aquaculture.

[44] Soonthornchai, W., Chaiyapechara, S., Jarayabhand, P., Soderhall, K. & Jiravanichpaisal, P. Interaction of *Vibrio* spp. with the inner surface of the digestive tract of *Penaeus monodon*. *PLoS One* **10**, e0135783, doi:10.1371/journal.pone.0135783 (2015). 10.1371/journal.pone.0135783 PMC4540450 26285030

[45] Björkroth, J. & Holzapfel, W. in *The prokaryotes* 267–319 (Springer, 2006).

[46] Farmer Iii, J. & Hickman-Brenner, F. in *The prokaryotes* 508–563 (Springer, 2006).

[47] Castex, M., Daniels, C. & Chim, L. Probiotic applications in crustaceans. *Aquaculture Nutrition: Gut Health, Probiotics and Prebiotics*, 290–327 (2014).

[48] Nam H, Ha M, Bae O, Lee Y (2002). Effect of *Weissella confusa* strain PL9001 on the adherence and growth of *Helicobacter pylori*. Appl Environ Microbiol.

[49] Cai Y, Benno Y, Nakase T, Oh T-K (1998). Specific probiotic characterization of *Weissella hellenica* DS-12 isolated from flounder intestine. J Gen Appl Microbiol.

[50] Alonso-Rodriguez R, Paez-Osuna F (2003). Nutrients, phytoplankton and harmful algal blooms in shrimp ponds: a review with special reference to the situation in the Gulf of California. Aquaculture.

[51] Kent M, Browdy CL, Leffler JW (2011). Consumption and digestion of suspended microbes by juvenile pacific white shrimp *Litopenaeus vannamei*. Aquaculture.

[52] Partensky, F., Blanchot, J. & Vaulot, D. Differential distribution and ecology of *Prochlorococcus* and *Synechococcus* in oceanic waters: a review. In *Marine cyanobacteria*, 457–476 (Monaco: Musée océanographique, 1999).

[53] Tai V, Palenik B (2009). Temporal variation of *Synechococcus* clades at a coastal Pacific Ocean monitoring site. ISME J.

[54] Martins R, Fernandez N, Beiras R, Vasconcelos V (2007). Toxicity assessment of crude and partially purified extracts of marine *Synechocystis* and *Synechococcus* cyanobacterial strains in marine invertebrates. Toxicon.

[55] Chen WY, Wu JH, Chang JE (2014). Pyrosequencing analysis reveals high population dynamics of the soil microcosm degrading octachlorodibenzofuran. Microbes Environ.

[56] Chen WY, Wu JH, Lin SC, Chang JE (2016). Bioremediation of polychlorinated-*p*-dioxins/dibenzofurans contaminated soil using simulated compost-amended landfill reactors under hypoxic conditions. J Hazard Mater.

[57] Edgar, R. C. UPARSE: highly accurate OTU sequences from microbial amplicon reads. *Nat Methods* **10**, 996-+, doi:10.1038/Nmeth.2604 (2013). 10.1038/nmeth.2604 23955772

[58] Magoč T, Salzberg SL (2011). FLASH: fast length adjustment of short reads to improve genome assemblies. Bioinformatics.

[59] Caporaso JG (2010). QIIME allows analysis of high-throughput community sequencing data. Nat Methods.

