# Supplementary material for: Microbiome Dynamics in a Shrimp Grow-out Pond with Possible Outbreak of Acute Hepatopancreatic Necrosis Disease
Source: Sci Rep. 2017 Aug 24;7:9395. doi: 10.1038/s41598-017-09923-6 (PMC5571196; doi:10.1038/s41598-017-09923-6)
Supplement: Supplementary file 1 — Supplementary Info [file 41598_2017_9923_MOESM1_ESM.pdf]

Supplementary Materials for the Manuscript

Microbiome Dynamics in a Shrimp Grow-out Pond with Possible Outbreak of Acute Hepatopancreatic Necrosis Disease

Wei-Yu Chen[1 *], Tze Hann Ng[2 *], Jer-Horng Wu[1 #], Jiung-Wen Chen[1], Han-Ching Wang[2 3 #]

[1]Department of Environmental Engineering, [2]Department of Biotechnology and Bioindustry Sciences, and [3]Center for Shrimp Disease Control and Genetic Improvement, National Cheng Kung University, Tainan 70101, Taiwan (ROC).

*WYC and THN share the first authorship.

[#]**Co-Corresponding author: Jer-Horng Wu**
E-mail address: enewujh@mail.ncku.edu.tw
Phone number: +886-6-2757575 ext.65839
[#]**Co-Corresponding author: Han-Ching Wang**
E-mail address: wanghc@mail.ncku.edu.tw
Phone number: +886-6-2757575 ext. 65603-810
Postal address: No.1, University Road, East District, Tainan City 701, Taiwan (R.O.C)
National Cheng Kung University (JHW: Department of Environmental Engineering; HCW: Department of Biotechnology and Bioindustry Sciences)

Table S1. Parameters of shrimp cultivation in the grow-out pond.

Items	Pond 1/A1
Area (m^2) × depth (m)	6,300×1.5
Postlarves (PL)	500,000
Density (PLs/m2)	79
Days of Cultivation (day)	38
Síze (pcs/kg)	363
Feed Total (kg)	1,390
Alive Shrimp Amount (kg)	1,229
Dead Shrimp Amount (kg)	60
Shrimp Total (kg)	1,289
Feed Conversion Ratio	1.08
Survival Rate (%)	94

Table S2. Screening of shrimp individuals with AHPND gene markers using a IQ real-time quantitative PCR assay.

		**IQ real (copies no./host genome)**	
#	Sample ID	Toxin1	AHPND plasmid
1	0913-C1	0	5.25E-06
2	0913-C2	0	2.95E-07
3	0913-C3	0	1.91E-05
4	0913-C4	0	1.31E-06
5	0915-C1	0	1.16E-05
6	0915-C2	0	9.57E-06
7	0915-C3	0	2.77E-05
8	0917-C1	0	2.42E-06
9	0917-C2	0	1.5E-07
10	0917-C3	0	8.97E-06
11	0923-C5	0	0
12	0925-C2	0	7.07E-06
13	0927-C1	0	0
14	0915-D2	7E-06	2.38E-06
15	0915-D4	5.98E-06	2.78E-06
16	0915-D5	3.38E-07	1.59E-06
17	0915-D6	1.62E-05	8.57E-07
18	0917-D2	1.86E-06	2.37E-06
19	0917-D3	6.11E-05	7.89E-05
20	0917-D4	5.26E-06	3.8E-06
21	0917-D5	1.01E-06	1.13E-06
22	0919-D1	3.84E-05	1.02E-05
23	0919-D3	3.73E-05	7.61E-06
24	0919-D5	2.04E-06	9.45E-06
25	0921-D1	7.51E-05	1.18E-05
26	0921-D2	6.97E-07	5.21E-06
27	0921-D3	8.07E-06	1.16E-06
28	0921-D4	2.17E-06	2.86E-06
29	0923-D1	1.47E-05	1.6E-05
30	0923-D3	3.59E-05	7.26E-06
31	0925-D1	9.55E-06	1.08E-05
32	0925-D3	5.13E-05	3.94E-05
33	0925-D5	1.48E-05	3.06E-06
34	0925-D7	3.67E-06	9.53E-07
35	0927-D1	0.000183	9.86E-06
36	0927-D2	1.82E-05	2.04E-06
37	0927-D4	1.58E-07	3E-06

Table S3. Read numbers and diversity indices of shrimp and pond seawater samples analyzed in this study.

#	Sample ID	Sample type	Chloroplast-free read (#)	Observed species	Shannon	Chao1	Avg Lgth (bp)
1	0913-C1	AHPND(-) Idvl	14000	694	6.41	853	410
2	0913-C2	AHPND(-) Idvl	14000	573	6.77	611	411
3	0913-C3	AHPND(-) Idvl	14000	707	7.45	797	412
4	0913-C4	AHPND(-) Idvl	14000	600	6.38	668	418
5	0915-C1	AHPND(-) Idvl	14000	681	6.39	842	413
6	0915-C2	AHPND(-) Idvl	14000	723	6.90	946	409
7	0915-C3	AHPND(-) Idvl	14000	627	5.88	749	413
8	0917-C1	AHPND(-) Idvl	14000	349	5.19	374	411
9	0917-C2	AHPND(-) Idvl	14000	579	6.96	671	411
10	0917-C3	AHPND(-) Idvl	14000	565	6.64	602	409
11	0923-C5	AHPND(-) Idvl	14000	638	6.17	858	409
12	0925-C2	AHPND(-) Idvl	14000	521	5.47	626	414
13	0927-C1	AHPND(-) Idvl	14000	428	5.48	475	411
14	0915-D2	AHPND(+) Idvl	14000	330	6.16	351	409
15	0915-D4	AHPND(+) Idvl	14000	743	6.91	865	409
16	0915-D5	AHPND(+) Idvl	14000	674	6.92	756	411
17	0915-D6	AHPND(+) Idvl	14000	689	6.89	817	409
18	0917-D2	AHPND(+) Idvl	14000	652	6.32	763	410
19	0917-D3	AHPND(+) Idvl	14000	446	5.53	650	407
20	0917-D4	AHPND(+) Idvl	14000	387	2.48	487	418
21	0917-D5	AHPND(+) Idvl	14000	620	6.11	709	408
22	0919-D1	AHPND(+) Idvl	14000	751	7.39	869	411
23	0919-D3	AHPND(+) Idvl	14000	410	5.98	432	414
24	0919-D5	AHPND(+) Idvl	14000	614	6.46	804	413
25	0921-D1	AHPND(+) Idvl	14000	522	5.15	658	417
26	0921-D2	AHPND(+) Idvl	14000	398	4.21	493	420
27	0921-D3	AHPND(+) Idvl	14000	100	1.51	184	426
28	0921-D4	AHPND(+) Idvl	14000	299	4.63	312	419
29	0923-D1	AHPND(+) Idvl	14000	361	3.78	482	417
30	0923-D3	AHPND(+) Idvl	14000	301	4.36	477	420
31	0925-D1	AHPND(+) Idvl	14000	142	2.00	273	424
32	0925-D3	AHPND(+) Idvl	14000	321	4.43	338	416
33	0925-D5	AHPND(+) Idvl	14000	245	2.35	337	423
34	0925-D7	AHPND(+) Idvl	14000	344	4.12	507	411
35	0927-D1	AHPND(+) Idvl	14000	268	3.58	461	418
36	0927-D2	AHPND(+) Idvl	14000	193	2.87	315	419
37	0927-D4	AHPND(+) Idvl	14000	153	2.10	263	425
38	initial-M	Pond Seawater	14000	443	5.26	711	411
39	0913-MA	Pond Seawater	14000	498	5.77	999	409
40	0913-MB	Pond Seawater	14000	625	5.79	1182	409
41	0913-MC	Pond Seawater	14000	549	5.74	1178	410
42	0915-MA	Pond Seawater	14000	596	5.98	1092	409
43	0915-MB	Pond Seawater	14000	489	5.72	1032	409
44	0915-MC	Pond Seawater	14000	615	5.94	1201	409
45	0917-MA	Pond Seawater	14000	590	5.86	1246	408
46	0917-MB	Pond Seawater	14000	553	5.81	1062	409
47	0917-MC	Pond Seawater	14000	612	6.03	1219	408
48	0919-MA	Pond Seawater	14000	398	5.23	721	409
49	0919-MB	Pond Seawater	14000	443	5.32	970	409
50	0919-MC	Pond Seawater	14000	419	5.30	817	408
51	0921-MA	Pond Seawater	14000	543	5.58	1047	408
52	0921-MB	Pond Seawater	14000	515	5.54	904	409
53	0921-MC	Pond Seawater	14000	513	5.57	952	408
54	0923-MA	Pond Seawater	14000	493	5.66	871	409
55	0923-MB	Pond Seawater	14000	505	5.52	952	408
56	0923-MC	Pond Seawater	14000	498	5.60	862	409
57	0925-MA	Pond Seawater	14000	514	5.93	932	410
58	0925-MB	Pond Seawater	14000	543	5.87	932	410
59	0925-MC	Pond Seawater	14000	572	5.93	1000	410
60	0927-MA	Pond Seawater	14000	539	6.02	909	410
61	0927-MB	Pond Seawater	14000	528	5.85	837	409
62	0927-MC	Pond Seawater	14000	556	5.98	929	409

Table S4. Primer sets used for PCR and qPCR in this study.

Target	Primer set	Sequence (5'-3')	Used for	Reference
Bacteria	27F<br>1492R	AGAGTTTGATCMTGGCTCAG<br>TACGGYTACCTTGTTACGACTT	PCR	[1]
Bacteria	1087F<br>1392R	GGTTAAGTCCCSYAACGAGC<br>ACGGGCGGTGTGTAC	qPCR	[2]
Archaea	A109F<br>1492R	ACKGCTCAGTAACACGT<br>TACGGYTACCTTGTTACGACTT	PCR	[3]
Archaea	Arc915F<br>Arc1059R	AGGAATTGGCGGGGGAGCAC<br>GCCATGCACCWCCTCT	qPCR	[4]
Bacteria	S17<br><br>A21	TCGTCGGCAGCGTCAGATGTGTATAAGAGA<br>CAGCCTACGGGNGGCWGCAG<br>GTCTCGTGGGCTCGGAGATGTGTATAAGAG<br>ACAGGACTACHVGGGTATCTAATCC	PCR/NGS	[5]

Reference:

[1] R.I. Amann, B.J. Binder, R.J. Olson, S.W. Chisholm, R. Devereux, D.A. Stahl, Combination of 16S rRNA-targeted oligonucleotide probes with flow cytometry for analyzing mixed microbial populations., Appl Environ Microbiol, 56 (1990) 1919-1925.

[2] W.Y. Chen, J.H. Wu, J.E. Chang, Pyrosequencing Analysis Reveals High Population Dynamics of the Soil Microcosm Degrading Octachlorodibenzofuran, Microbes and environments / JSME, (2014).

[3] R. Grosskopf, P.H. Janssen, W. Liesack, Diversity and structure of the methanogenic community in anoxic rice paddy soil microcosms as exam- ined by cultivation and direct 16S rRNA gene sequence retrieval, Appl Environ Microbiol, 64 (1998) 960-969.

[4] C. Lee, J. Kim, S.G. Shin, S. Hwang, Monitoring bacterial and archaeal community shifts in a mesophilic anaerobic batch reactor treating a high-strength organic wastewater, FEMS Microbiol Ecol, 65 (2008) 544-554.

[5] A. Klindworth, E. Pruesse, T. Schweer, J. Peplies, C. Quast, M. Horn, F.O. Glockner, Evaluation of general 16S ribosomal RNA gene PCR primers for classical and next-generation sequencing-based diversity studies, Nucleic Acids Res, 41 (2013).

Figure S1 (Chen et al., 2017)

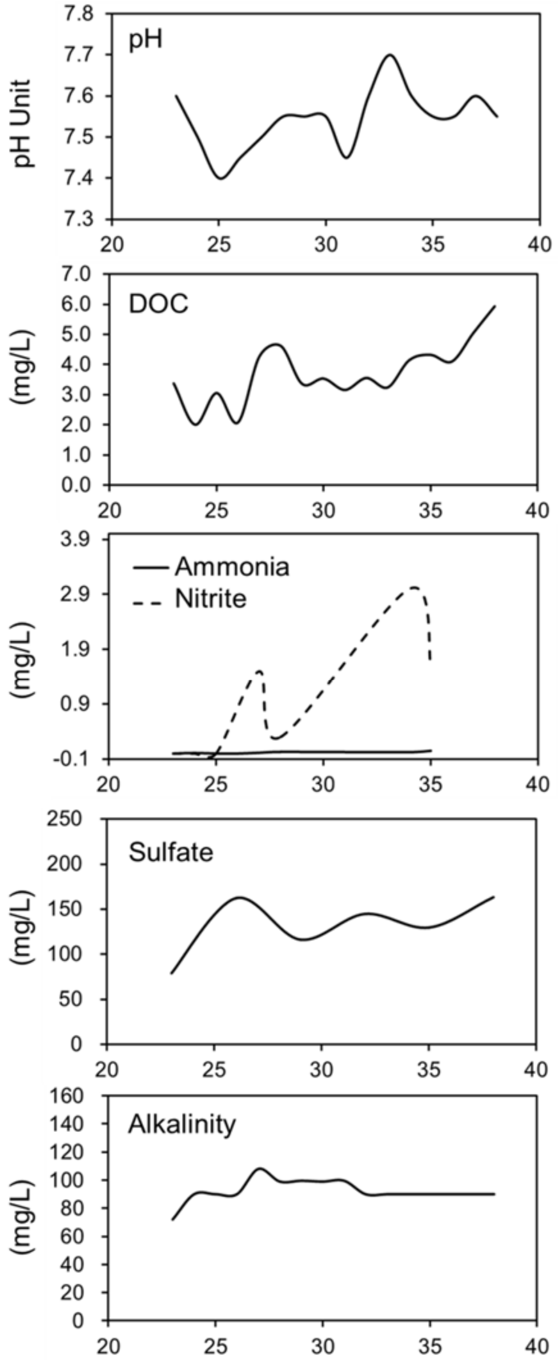

Figure S1. Concentration variations of hydrogen ion (pH), dissolved organic carbon (DOC), ammonia, nitrite, sulphate, and alkalinity in seawater from the shrimp cultivation pond during the sampling periods. pH, ammonia, and nitrite were measured on site by using a portable pH meter (SP-701, Suntex, Taiwan), an ammonia-salicylate reagent (Method 8155, Hach), and a NitriVer nitrite reagent (Method 8507, Hach) kit, respectively, whereas alkalinity was analysed according to Standard Methods. Prior to the analysis of DOC and sulphate, samples of 45 mL were filtered through a 0.45-μm disposable membrane and analysed using an InnovOx TOC analyser (General Electric, USA) and ion chromatography (Dionex ICS-1100, Thermo Fisher, USA)

Figure S2 (Chen et al., 2017)

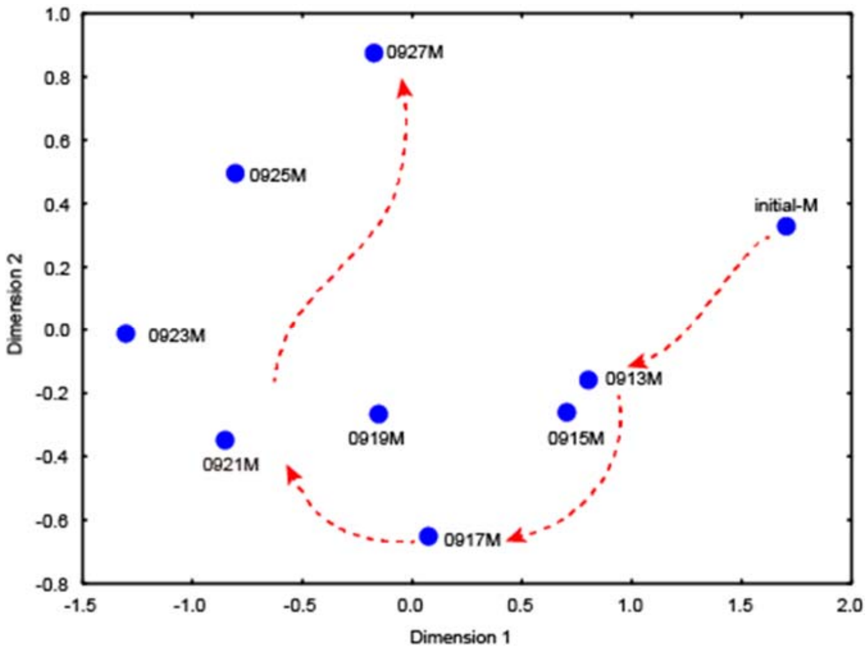

Figure S2. Euclidean distance-based nonmetric multidimensional scaling (NMDS) plot of bacterial community compositions in pond seawater.

Figure S3 (Chen et al., 2017)

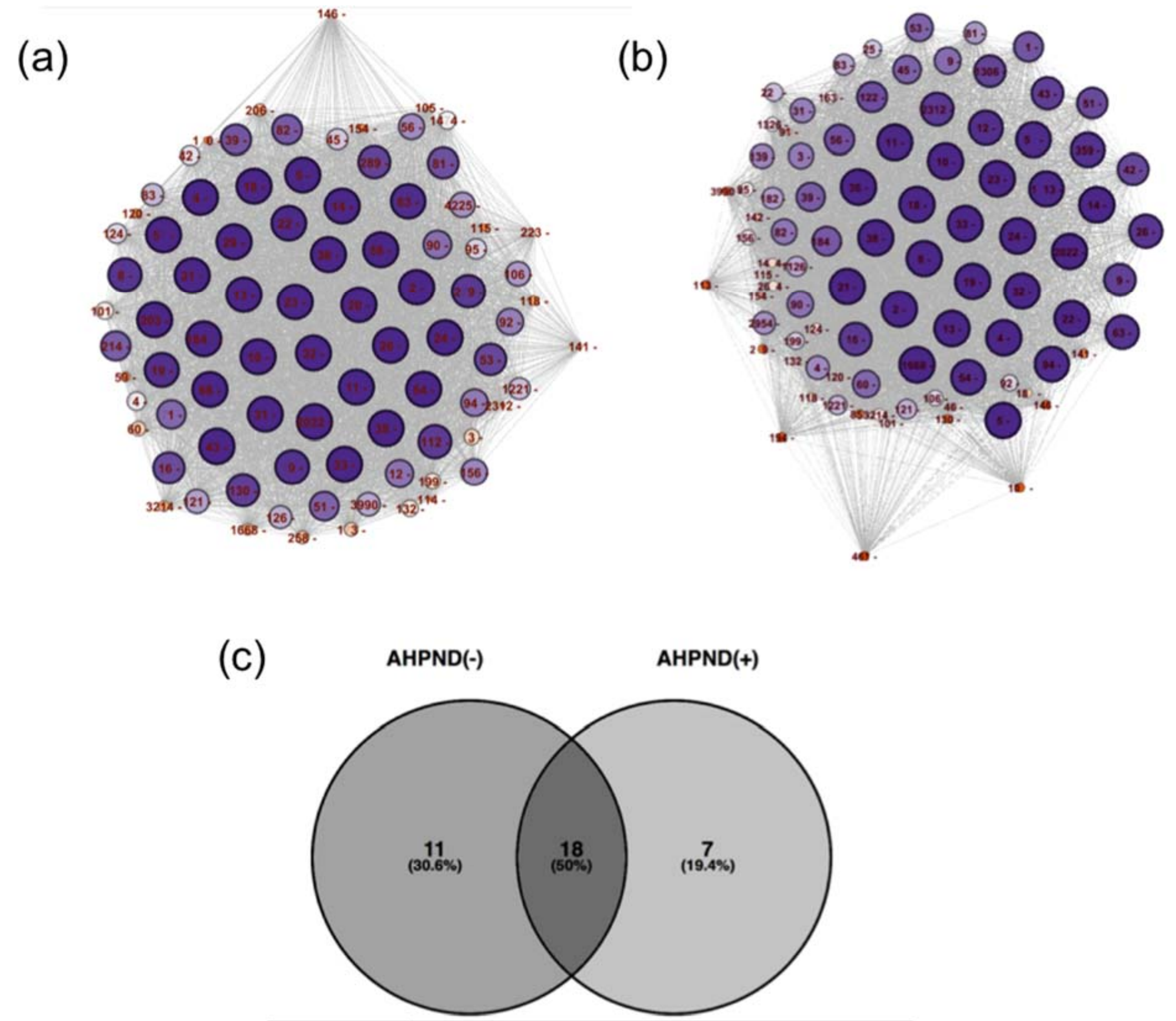

	OTU number	
Shared	Bacilloplasma: 2, 4, 5<br>Cyanobacteria_I: 22, 2022<br>Rhodobacteraceae: 10<br>Synechococcus:11<br>Pirellula: 13<br>Blastopirellula:14<br>γ-proteobacteria: 18<br>Anderseniella: 21	Actinobacteria: 23<br>Sphingomonas: 24<br>Micrococcales: 32<br>Weissella: 33<br>Planctomycetaceae: 36<br>Aegilops: 38<br>Caldilineaceae: 54
AHPND(-) only	Vibrio: 20<br>Waddlia: 26<br>γ-proteobacteria: 29, 1847<br>Bradyrhizobium: 31<br>Shewanella: 43<br>Mycoplasmataceae: 57	Clostridium sensu stricto 17: 58<br>Rhodobacteraceae: 63<br>Ralstonia: 68<br>Rickettsiales: 203
AHPND(+) only	Vibrio: 8, 1668, 1713<br>Microbacteriaceae: 12	PeM15: 19<br>Acinetobacter: 53, 94

Figure S3. Microbial consensus networks in **(A)** AHPND(−) and **(B)** AHPND(+) shrimp stomach communities. Nodes (circle) depict the microbial populations (OTUs), and node colours (size) indicate the interactive strength. **(C)** Venn diagram analysis depicting the numbers of shared and unique OTUs with the most frequent interactive relationships (large purple circle) from AHPND(−) and AHPND(+) samples. The taxonomic information of each OTU is tabulated below the Venn diagram.

Figure S4 (Chen et al., 2017)

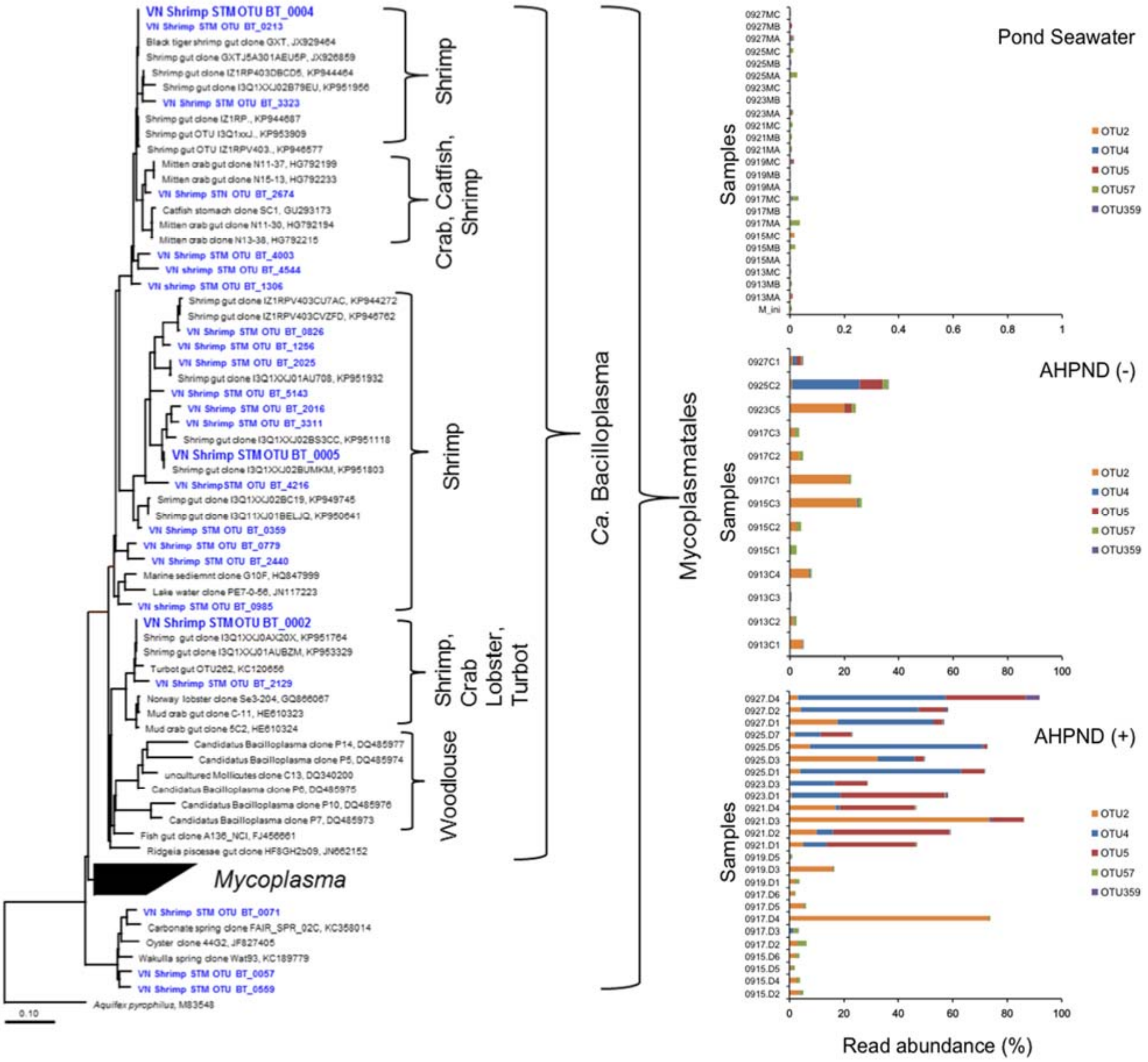

Figure S4. Phylogenetic tree of Mycoplasmatales-related 16S rRNA gene sequences retrieved in this study (in blue boldface) and their relatives from the SILVA database (https://www.arb-silva.de/). The sequences of the selected OTUs were added to the SSURef guide tree by using a parsimony method provided in a ARB package. The phylogenetic tree with *Aquifex pyrophilus* (M83548) as the outgroup was then reconstructed with the same sequence region by using the neighbour-joining algorithm. The scale bar corresponds to 10 nucleotide substitutions per 100 nucleotides. The sequence read abundances of abundant OTUs from Mycoplasmatales in pond seawater, AHPND(−), and AHPND(+) samples are displayed, respectively, on the right side of the tree.

Figure S5 (Chen et al., 2017)

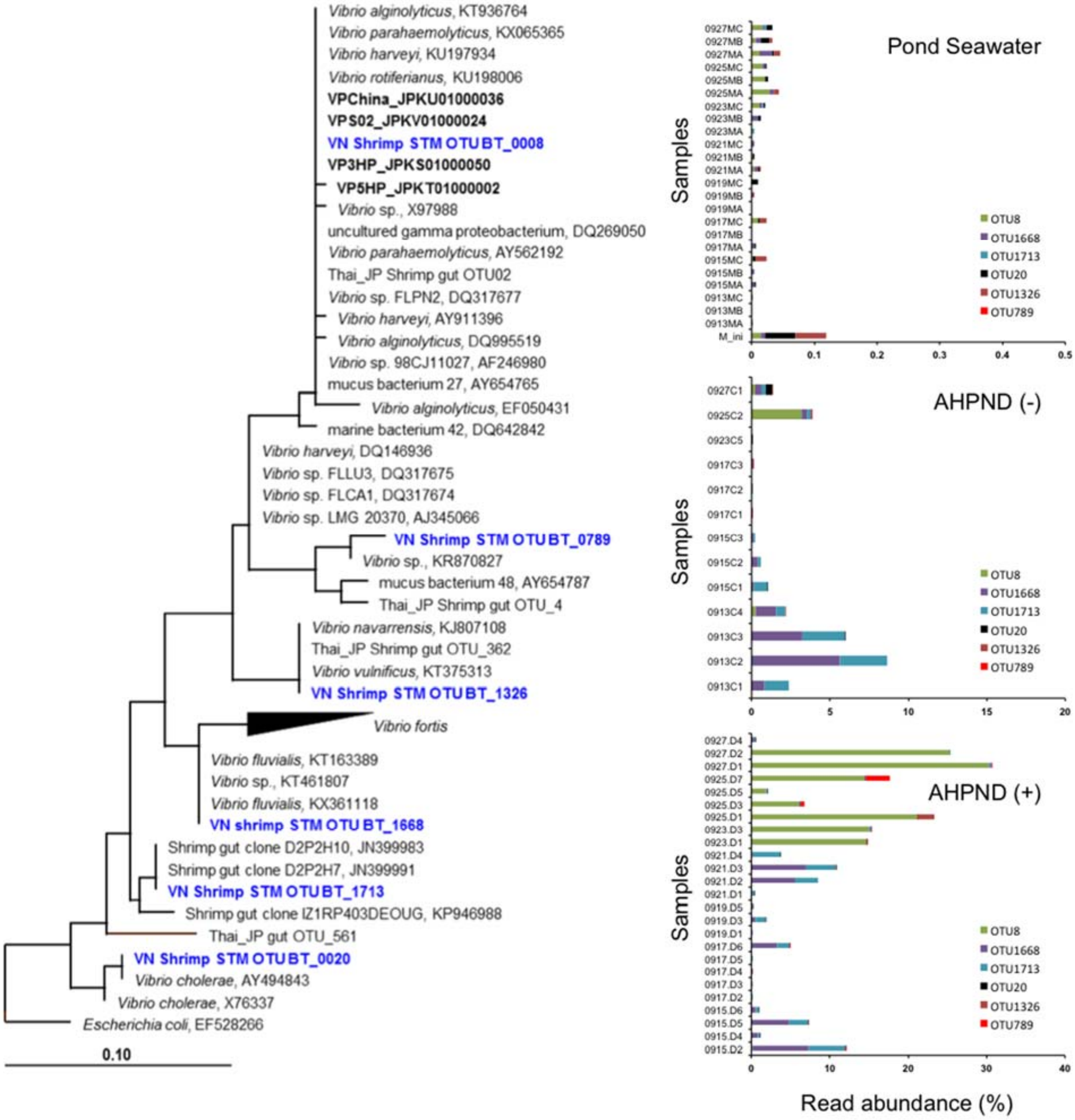

Figure S5. Phylogenetic tree of *Vibrio*-related 16S rRNA gene sequences retrieved in this study (in blue boldface) and their relatives from the SILVA database (https://www.arb-silva.de/). The sequences of the selected *Vibrio* OTUs were added to the SSURef guide tree by using a parsimony method provided in an ARB package. The phylogenetic tree with *Escherichia coli* (EF528266) as the outgroup was then reconstructed with the same sequence region by using the neighbour-joining algorithm. The scale bar corresponds to 10 nucleotide substitutions per 100 nucleotides. The sequence read abundances of abundant *Vibrio* OTUs in pond seawater, AHPND(−), and AHPND(+) samples are displayed, respectively, on the right side of the tree.
